# Supplementary material for: Drug repurposing for aging research using model organisms
Source: Aging Cell. 2017 Jun 16;16(5):1006–15. doi: 10.1111/acel.12626 (PMC5595691; doi:10.1111/acel.12626)
Supplement: Supplementary file 7 — Data S1 Zip‐Archive of all report cards. [file ACEL-16-1006-s007.zip › RC_ZYS.pdf]

ZYS

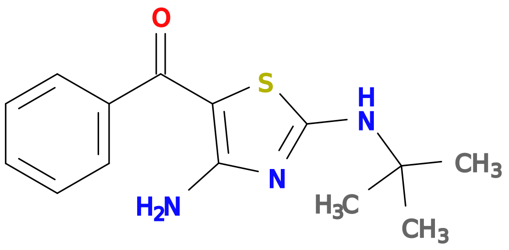

Database identifiers

ChEMBLCompound	CHEMBL570537
DrugBank	DB08778
ZINC	ZINC01398629
eMolecules	36800750

Ranking

	Rank	Score
Drosophila	382/697	0.378
C. elegans	190/591	0.254

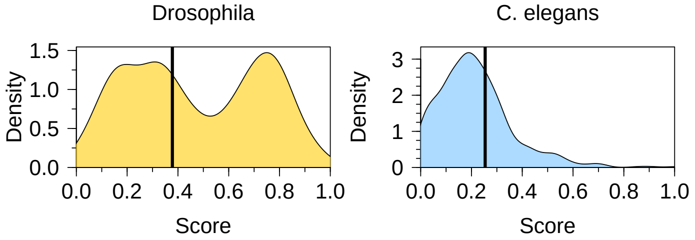

	Ageing implication	Domain conservation	Binding site conservation	Binding affinity	Bioavailability	Lipinski	Promiscuity	Purchasability	Drug approval	**Total**
Drosophila	0.563	0.939	0.773	0.554	(0.9)	0.0	-0.0	0.1	0.075	0.378
C. elegans	0.563	0.885	0.773	0.554	0.373	0.0	-0.0	0.1	0.075	0.254

Names

No synonyms found

Roles

ChEBI entry None has no roles

Status

Approved drug (according to ChEMBL)	No
Classification (according to DrugBank)	experimental
Number of Rule of 5 violations	0
Binding affinity to original target in log units (RF-Score prediction)	5.22
Burns *C. elegans* bioavailability prediction	1.1

Compound Target Characteristics

Serine/threonine-protein kinase Chk1

Best gene implication in ageing for this target family came from gene O14757 annotated in UniProt release 2014_02. Annotation GO subterm of 7568 (aging) was Non-traceable Author Statement

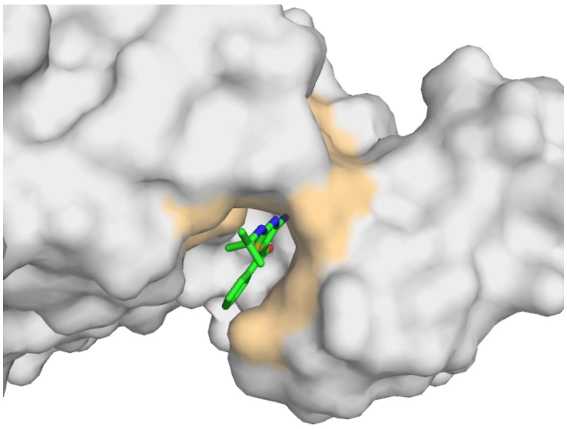

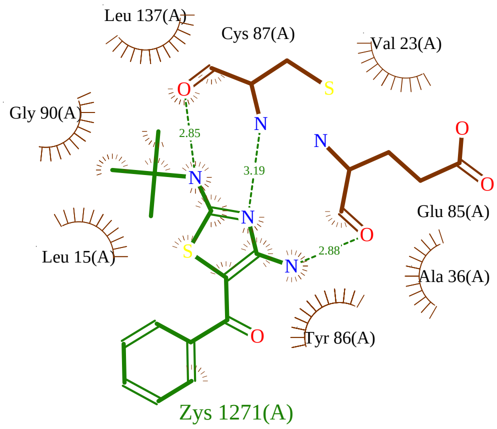

protein	amino acids contacts (binding site)
PDB:2wms:chainA:O14757	L V A E Y C G L
tr:E9PJI4:E9PJI4_HUMAN	L V A E Y C G L
tr:E9PKQ3:E9PKQ3_HUMAN	L V A E Y C G L
tr:J3KN87:J3KN87_HUMAN	L V A E Y C G L
sp:O14757:CHK1_HUMAN	L V A E Y C G L
sp:Q91ZN7:CHK1_RAT	L V A E Y C G L
tr:G3UYB1:G3UYB1_MOUSE	L V A E Y C G L
tr:G3UYC5:G3UYC5_MOUSE	L V A E Y C G L
sp:O35280:CHK1_MOUSE	L V A E Y C G L
sp:O61661:CHK1_DROME	L V A E Y A G L
tr:Q965F6:Q965F6_CAEEL	L V A E Y A G L
tr:O44547:O44547_CAEEL	L V A E Y A G L
sp:Q9N3Z3:CHK1_CAEEL	L V A E Y A G L
sp:P38147:CHK1_YEAST	V V A E M A G L

	whole protein		domain-based		contact-based	
protein	ident	simil	ident	simil	ident	simil
PDB:2wms:chainA:O14757	1.0	1.0	1.0	1.0	1.0	1.0
tr:E9PJI4:E9PJI4_HUMAN	0.36	0.36	0.63	0.63	1.0	1.0
tr:E9PKQ3:E9PKQ3_HUMAN	0.43	0.43	0.76	0.76	1.0	1.0
tr:J3KN87:J3KN87_HUMAN	0.91	0.91	1.0	1.0	1.0	1.0
sp:O14757:CHK1_HUMAN	1.0	1.0	1.0	1.0	1.0	1.0
sp:Q91ZN7:CHK1_RAT	0.94	0.98	0.98	1.0	1.0	1.0
tr:G3UYB1:G3UYB1_MOUSE	0.84	0.89	0.97	0.99	1.0	1.0
tr:G3UYC5:G3UYC5_MOUSE	0.87	0.91	0.97	0.99	1.0	1.0
sp:O35280:CHK1_MOUSE	0.93	0.98	0.97	0.99	1.0	1.0
sp:O61661:CHK1_DROME	0.44	0.75	0.56	0.87	0.88	0.77
tr:Q965F6:Q965F6_CAEEL	0.2	0.4	0.37	0.73	0.88	0.77
tr:O44547:O44547_CAEEL	0.19	0.41	0.35	0.74	0.88	0.77
sp:Q9N3Z3:CHK1_CAEEL	0.3	0.63	0.48	0.8	0.88	0.77
sp:P38147:CHK1_YEAST	0.23	0.62	0.35	0.74	0.63	0.69

grp (FBgn0261278) associated phenotypes

cell cycle defective, chemical sensitive, decreased cell death, maternal effect, mitotic cell cycle defective, non-rescuable maternal effect, partially lethal - majority die, radiation resistant, radiation sensitive

(Information from FlyBase)

grp (UniProt:O61661) annotation
Function: Serine/threonine-protein kinase which is required for checkpoint-mediated cell cycle arrest and activation of DNA repair in response to the presence of DNA damage or unreplicated DNA. May also negatively regulate cell cycle progression during unperturbed cell cycles. May phosphorylate the CDC25 phosphatase stg, which promotes its degradation. This results in increased inhibitory tyrosine phosphorylation of CDC2-cyclin complexes and consequent inhibition of cell cycle progression. (PubMed:10209095, PubMed:10469601, PubMed:10980701, PubMed:12919679, PubMed:14711410, PubMed:15723794, PubMed:15860729, PubMed:16079276, PubMed:7925016, PubMed:9197245, PubMed:9214509).
Subcellular location: Nucleus ECO:0000269—PubMed:10980701, PubMed:16079276).
Developmental stage: Expressed both maternally and zygotically. Maternally supplied mRNA is degraded during progression from nuclear stage 12 to nuclear stage 13. Zygotic expression is seen at reduced levels later in embryogenesis and during larval development. Higher expression is seen in pupae, coincident with ovarian differentiation. May be activated during the syncytial blastoderm divisions which precede cellularization, the Drosophila equivalent of the midblastula transition (MBT). Developmentally regulated activation of the DNA replication checkpoint may occur as the nucleo-cytoplasmic ratio increases and maternal replication factors are depleted. Elongation of the embryonic cell cycle may allow time for the transcription of genes that initiate the switch from maternal to zygotic control of embryogenesis. (PubMed:9197245, PubMed:9214509).
Ptm: Phosphorylated in a MEI-41/ATR dependent manner in response to DNA damage or the presence of unreplicated DNA. (PubMed:15860729).

(Information from UniProt)

chk-1 (WBGene00000498) associated phenotypes
lethal, locomotion variant, mid larval lethal, sterile

(Information from WormBase)

chk-1 (UniProt:Q9N3Z3) annotation
Function: Serine/threonine-protein kinase which is required for checkpoint-mediated cell cycle arrest and activation of DNA repair in response to the presence of DNA damage or unreplicated DNA. May also negatively regulate cell cycle progression during unperturbed cell cycles. Required for checkpoint mediated cell cycle arrest in response to DNA damage in germline cells. Essential for embryogenesis. (PubMed:15326393).
Subcellular location: Cytoplasm Nucleus
Tissue specificity: Expressed in the germline. (PubMed:15326393).
Developmental stage: Highly expressed in the embryo. (PubMed:15326393).

(Information from UniProt)
